# Oral Health Status and Treatment Needs Among Disabled Children in Recife, Brazil

**DOI:** 10.3290/j.ohpd.a44030

**Published:** 2020-07-04

**Authors:** Ângela Maria Brito Ferreira, Híttalo Carlos Rodrigues de Almeida, Mônica Vilela Heimer, Sandra Conceição Maria Vieira, Viviane Colares

**Affiliations:** a PhD Student, Pediatric Dentistry, School of Dentistry, University of Pernambuco – FOP/UPE, Camaragibe-PE, Brazil. Contributed to article selection, data analysis and interpretation, data collection and manuscript draft.; b MSc Student, School of Dentistry, University of Pernambuco – FOP/UPE, Camaragibe-PE, Brazil. Contributed to data collection, data analysis and interpretation, and manuscript drafting.; c Master’s Student, Graduate Program in Adolescent Health, School of Dentistry, University of Pernambuco – FOP/UPE, Camaragibe-PE, Brazil. Contributed to the analysis, interpretation of data and revision of the manuscript.; d Master’s Student, Graduate Program in Adolescent Health, School of Dentistry, University of Pernambuco – FOP/UPE, Camaragibe-PE, Brazil. Contributed to the manuscript review.; e Master’s Student, Graduate Program in Pediatric Dentistry, School of Dentistry, University of Pernambuco – FOP/UPE, Camaragibe-PE, Brazil. Contributed to data analysis and interpretation, and manuscript revision.

**Keywords:** children, dental care, developmental disabilities, oral health

## Abstract

**Purpose:**

The objective of the present study was to investigate oral health status and treatment needs of children with disabilities in Recife, Brazil.

**Materials and Methods:**

A cross-sectional study was carried out in the six administrative districts of Recife. The sample consisted of 366 children with disabilities and age between 3 and 12 years. The oral health conditions investigated were dental caries (CPOD index and dmft index), gingival state (IPV and IGC index) and dental trauma. Data analysis involved descriptive statistics, Pearson’s chi-square test, Fisher’s exact test, and Poisson regression models.

**Results:**

The prevalence of caries was 65% and was associated with age (p = 0.0027) and area of residence (p = 0.020). The prevalence of need for treatment was also 65%. The mean decayed, missing and filled teeth (DMFT)/DMFT index of the study population was 3.17/1.73. Their mean number of DMFT was 2.37, 0.55 and 0.25 for the deciduous dentition, as well as 1.56, 0.05 and 0.12 for the permanent dentition. Almost the entire sample (96.7%) had visible plaque, 77.3% had gingival bleeding and 27.6% had dental traumatism.

**Conclusion:**

Children with disabilities were found to have high rates of caries and gingivitis, as well as cumulative needs for preventive and curative treatment.

The American Health Association defines a child with disability as one who, for various reasons, cannot fully make use of all his/her physical, mental and social abilities.^4^ In the context of dentistry, children with disabilities constitute a segment of patients regarded as special, because, due to their deviation from the standard of normality, they require special attention and specific approaches during a certain period of their lives or indefinitely.^24^

According to reports in the literature, disabled children often have worse oral health status than the general population. They tend to have a high prevalence of dental caries and difficulty in gaining access to dental care.^4,11,16^ Studies have shown that oral health challenges are more complex in disabled children, who are unable to adequately apply the techniques necessary for plaque control.^4,10^ In most cases, the responsibility for the oral hygiene of a disabled child lies with another person, generally a parent or caregiver, many of whom are emotionally or intellectually incapable of dealing with the health problems of their less fortunate young ones.^2,8,19,22^

The aim of the present study was to investigate the oral health status and treatment needs of children with disabilities and determine associated factors.

## Materials and Methods

**Fig 1:**
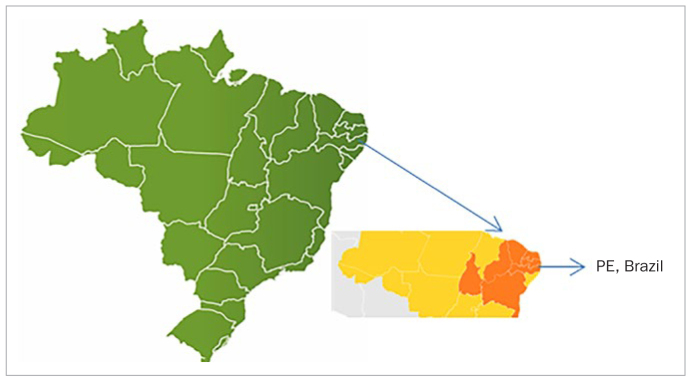
The city of Recife, PE, Brazil.

This study was conducted in the six administrative districts that make up the Public Health Department of the city of Recife, PE, Brazil (Fig 1). The sample consisted of 366 children with disabilities aged 3–12 years, representative of the population of children with disabilities enrolled in the Family Health Program, which is a branch of primary care offered by the Brazilian federal government.

As there are different prevalence rates for the different oral health conditions analysed in the present study, a 50% prevalence rate was considered for the sample calculation to maximise the size and improve the statistical power of the findings.

The oral health conditions investigated were dental caries and treatment needs (decayed, missing and filled teeth [DMFT/dmft] index),^17^ gingival status (visible plaque index [VPI] and gingival bleeding index [GBI])^1^ and dental trauma (Andreasen, 2001).^5^ Information on oral health status and treatment needs was collected using the WHO form. Personal data, socioeconomic characteristics and issues related to access to oral health services were investigated using a form validated by Aragão et al (2011).^6^ The research team was composed of two people (examiner and annotator) who had undergone training and calibrations exercises for the standardisation of the examining techniques, as described in the oral health survey manual. Intraexaminer Kappa coefficients were determined for dental caries (k = 0.93), visible plaque (k = 0.91), bleeding gums and dental trauma (k = 0.97).

A database was constructed and the data were analysed using the SPSS 17.0 software. Data analysis involved descriptive statistics, Pearson’s chi-square test, Fisher’s exact test and Poisson regression models, with a p value ≤ 0.05 indicative of statistical significance.

This study received approval from the Human Research Ethics Committee of the University of Pernambuco, Brazil (certificate number: 187/09). The data were collected following authorisation from the municipal health department and the voluntary signing of an informed consent form by the adult responsible for the child.

## Results

A total of 366 children (206 boys [56.3%] and 160 girls [43.7%]) were examined. The largest proportion (49.5%) of the children was between 5 and 9 years old. A total of 47.8% of the children had mental disabilities, 13.9% had physical disabilities, 29% had multiple disabilities and 9.3% had sensory impairment (7.4% hearing and 1.9% visual impairment). Most of the children (76.2%) attended school, but a mastery of reading and writing was seen in only 13.7% and 17.8%, respectively. Among the guardians, the most prevalent level of schooling (46.2%) was an incomplete primary education. In most cases, the mother was the caregiver (82.2%) and a housewife (82.5%) with a low education level (82.5%). Family income was less than three times the Brazilian monthly minimum wage (Table 1).

**Table 1:** Distribution of children analysed according to age group, gender, socioeconomic and demographic characteristics

Variables	n	%
TOTAL	366	100.0
**Age group (years)**		
3 < 5	74	20.2
5 < 9	181	49.5
9 to 12	111	30.3
**Gender**		
Boys	206	56.3
Girls	160	43.7
**Attending school**		
Yes	279	76.2
No	87	23.8
**Child’s parent or guardian**		
Mother	301	82.2
Father	16	4.4
Grandmother	34	9.3
Other	15	4.1
**Parent’s/guardian’s level of education**		
Illiterate	9	2.5
Incomplete elementary education	169	46.2
Complete elementary/incomplete high school	116	31.7
Complete high school	58	15.8
University – incomplete or complete	14	3.8
**Parent’s/guardian’s occupation**		
Housewife	302	82.5
Informal employment	18	4.9
Day labourer	16	4.4
Retiree/Pensioner	12	3.3
Security guard	10	2.7
Other	8	2.1
**Family income**		
< 1 Brazilian monthly minimum wage	17	4.6
1 to < 2 times Brazilian monthly minimum wage	151	41.3
2 or more times Brazilian monthly minimum wage	198	54.1

Table 2 displays the results of the Poisson regression for the prevalence of caries. Statistically significant associations were found for the number of residents in the home and the place of resident. The probability of having caries increased with the increase in the number of the residents and was higher in districts II and I.

**Table 2:** Multivariate analysis of associations between caries and sociodemographic data related to child and guardian

Variables	Caries				
	n	Crude PR (95% CI)	P value	Adjusted PR (95% CI)	P value
**Age group (years)**					
3 < 5	40	1.00	p = 0.0027*	1.00	p = 0.314
5 < 9	113	1.15 (0.91–1.47)		1.12 (0.88–1.41)	
9 to 12	81	1.35 (1.06–1.71)		1.20 (0.94–1.52)	
**Residents at home**					
2 to 3	53	1.00	p = 0.009*	1.00	p = 0.002*
4 to 6	121	1.23 (0.99–1.52)		1.30 (1.06–1.60)	
7 or more	60	1.42 (1.13–1.77)		1.50 (1.19–1.87)	
**Schooling of guardian (years of study)**					
< 8	123	1.32 (1.03–1.68)	p = 0.048*	1.29 (1.01–1.65)	p = 0.078
8 complete	74	1.22 (0.94–1.59)		1.11 (0.85–1.44)	
> 8	37	1.00		1.00	
**Guardian works outside home**					
Yes	29	1.00	p = 0.186	1.00	p = 0.452
No	215	1.17 (0.91–1.30)		0.99 (0.77–1.27)	
**Place of residence**					
District I	42	1.38 (1.55–1.66)	p = 0.001*	1.59 (1.26–2.00)	P < 0.0011*
District II	12	1.41 (1.09–1.35)		1.50 (1.15–1.94)	
District III	40	1.04 (0.82–1.32)		0.99 (0.79–1.24)	
District IV	20	0.71 (0.50–1.02)		0.78 (0.55–1.11)	
District V	44	1.06 (0.85–1.33)		1.12 (0.89–1.4)	
District VI	76	1.00		1.00	

(* ) statistically significant at 5% level.

Table 3 displays the results of the Poisson regression for the prevalence of bleeding gums, which was associated with age. The probability of exhibiting gingivitis was higher in the older age group. The prevalence of dental trauma was associated with age and family income (Table 4).

**Table 3:** Multivariate analysis of associations between gingival bleeding and sociodemographic data related to child and guardian

Variables	Gingival bleeding				
	n	Crude PR (95% CI)	P value	Adjusted PR (95% CI)	P value
**Age group (years)**					
3 < 5	50	1.00	p < 0.001*	1.00	p = 0.001*
5 < 9	133	1.09 (0.91–1.30)		1.06 (0.89–1.27)	
9 to 12	100	1.33 (1.13–1.58)		1.30 (1.10–1.54)	
**Residents in home**					
2 to 3	75	1.00	p = 0.097*	1.00	p = 0.071
4 to 6	139	1.23 (0.99–1.52)		1.30 (1.06–1.60)	
7 or more	69	1.42 (1.13–1.77)		1.50 (1.19–1.87)	
**Guardian works outside home**					
Yes	33	1.00	p = 0.010*	1.00	p = 0.056
No	250	1.25 (1.01–1.55)		1.22 (0.99–1.50)	
**Type of disability**					
Physical	33	1.00	p = 0.107	1.00	p = 0.266
Mental	136	1.20 (0.97–1.49)		0.98 (0.81–1.19)	
Sensory	27	1.23 (0.94–1.60)		0.81 (0.62–1.05)	
Multiple	87	1.27 (1.02–1.58)		1.00 (0.82–1.22)	

(* ) statistically significant at 5% level.

**Table 4:** Multivariate analysis of associations between dental trauma and sociodemographic data related to child and guardian

Variables	Dental trauma				
	n	Crude PR (95% CI)	P value	Adjusted PR (95% CI)	P value
**Age group (years)**					
3 < 5	37	3.47 (2.09–5.76)	p < 0.001*	3.22 (1.95–5.31)	p < 0.001*
5 < 9	48	1.84 (1.10–3.08)		1.76 (1.06–2.91)	
9 to 12	16	1.00		1.00	
**Gender**					
boys	63	1.29 (0.91– 1.82)	p = 0.47	1.20 (0.86–1.67)	p = 0.277
girls	38	1.00		1.00	
**Residents at home**					
2 to 3	29	1.55 (0.89–2.68)	p = 0.129	1.05 (0.61–1.83)	p = 0.356
4 to 6	57	1.63 (0.99–2.71)		1.30 (0.78–2.18)	
7 or more	15	1.00		1.00	
**Family income (Brazilian monthly minimum wage)**					
≥ 2 times	60	1.72 (1.23–2.42)	p = 0.001*	1.42 (1.00–2.00)	p = 0.049*
> 2 times	41	1.00		1.00	
**Place of residence**					
District I	19	1.36 (0.86–2.13)	p = 0.036*	1.27 (0.83–1.95)	p = 0.055
District II	5	1.28 (0.60–2.72)		1.26 (0.62–2.57)	
District III	11	0.62 (0.34–1.14)		0.64 (0.36–1.14)	
District IV	18	1.40 (0.88–2.21)		1.01 (0.64–1.57)	
District V	13	0.68 (0.39–1.20)		0.56 (0.33–0.97)	
District VI	35	1.00		1.00	

(* ) statistically significant at 5% level.

Regarding caries experience in the primary dentition, the children had a mean dmft index of 3.17, with a mean of 2.37 on the decayed component alone. In the permanent dentition, the children had a mean DMFT index of 1.73, with a mean of 1.56 on the decayed component alone. The decayed component was the highest percentages in both the dmft and DMFT indexes (74.7% and 90.2%, respectively). A total of 65% of the sample had treatment needs (Table 5).

**Table 5:** Distribution of treatment needs due to caries by children and tooth evaluated

Variable	n	%
**Treatment needs of children**		
No	128	35.0
Yes	238	65.0
**Total**	**366**	**100.0**
**Treatment needs of children**		
Filling of one surface	199	84.3
Filling of two or more surfaces	62	26.3
Esthetic facet	8	3.3
Pulp therapy plus restoration	22	9.3
Teeth indicated for extraction	48	20.3
Remineralization of white spot	53	22.5
Sealant	30	12.7
BASELINE^(1)^	238	-
**Treatment needs by tooth**		
No Yes	7007 1345	83.9 16.1
**Total** ^(2)^	**8352**	**100.0**
**Treatment needs by tooth**	**n**	**%**
Filling of one surface	718	8.6
Filling of two or more surfaces	161	1.9
Esthetic facet	16	0.2
Pulp therapy plus restoration	35	0.4
Teeth indicated for extraction	96	1.2
Remineralization of white spot	179	2.1
Sealant	140	1.7

^(1)^ As a child could have more than one treatment need, only the baseline records were used for the calculation of percentage. ^(2)^ Among the 8352 evaluated teeth, 4643 were primary teeth and 3709 were permanent teeth.

## Discussion

Good health is a fundamental goal for people and the society in which they live.^2,3^ We found that children with disabilities have high rates of dental caries and poor oral hygiene. Research shows that poor oral hygiene in children with disabilities affects chewing, nutrition, speech and quality of life.^9,18^

Dental caries has a multifactor aetiology involving a combination of primary factors (microbiota, host and substrate) and social factors.^21^ Aspects related to the socioeconomic status of the population, such as schooling, income, social class and behaviours, should be analysed when analysing the development of this oral problem.^23^

In the present study, the prevalence of dental caries was high among the children investigated, which seems to suggest the difficulty health services have in addressing contextual risk factors as well as implementing preventive and curative policies in primary oral healthcare for the child population with disabilities. Similar results are reported in previous studies.^14,20^

The prevalence of caries increased significantly with age, which is in agreement with data described in previous studies that investigated the influence of age on the development of dental caries in children with disabilities.^4,15,23^ A possible explanation for this increase may be related to the accumulation of sociobiological risks acting continuously with the advance in age.^9^

Studies have shown that children with poor nutrition have poor oral hygiene.^2,11,13^ In the present study, the children were from low-income families, the majority of which earned less than three times the Brazilian monthly minimum wage. The investigation of factors such as the parent’s/guardian’s level of education, child’s gender, age and socioeconomic status is important to the study of oral conditions, as it enables the establishment of more effective prevention measures.^9,18^

The prevalence of gingivitis was high (77.3%), especially among children with multiple disabilities, although no statistically significant association was found. These findings are in line with data described by Brown and Schodel (2014),^7^ who report that children with disabilities have poorer oral hygiene compared to non-disabled children. High frequencies of gingivitis have also been found in other studies.^8,16^ Their authors are unanimous in reporting that brushing is the main and most effective measure for plaque control and the prevention of periodontal diseases and, due to the limitations of this specific population, the lack of brushing explains the high rates of gingivitis. The use of medicine, which is often a part of these children’s lives, may be a contributing factor in this respect. Moreover, gingivitis was associated with age in the present sample, which has also been reported in previous studies.^4,8^

The prevalence of dental trauma is in agreement with findings described by DeMattei (2007)^8^ and was associated with age and family income. The occurrence of dental trauma in children occurs mainly at school or home, with a higher incidence at the age of 3 years involving the maxillary anterior teeth, especially the central incisors.^15^ The age of the greatest occurrence of dental trauma is early childhood, with a higher prevalence among boys.^12^

## Conclusion

The present findings can be explained by the difficulty children with disabilities have in gaining access to dental services, which makes it difficult to obtain information regarding prevention, care and the importance of maintaining one’s oral health.^3^ All these factors are aggravated by an underprivileged socioeconomic background and the sociobiological risks to which many children with disability are exposed.^15^

Therefore, there is a need for an oral health programme that emphasises prevention, leading to an urgent improvement in the training of dentists for the effective treatment of children with disabilities and also for the training of caregivers of these children.
